# A comparison between the Mini-Mental State Examination and the Montreal Cognitive Assessment Test in schizophrenia

**DOI:** 10.4102/sajpsychiatry.v22i1.890

**Published:** 2016-05-06

**Authors:** Mia Rademeyer, Pierre Joubert

**Affiliations:** 1 Department of Psychiatry, School of Medicine, University of Pretoria, South Africa

## Abstract

**Background:**

Cognitive impairment is a core feature of schizophrenia that also has strong prognostic significance. In most clinical settings comprehensive neuropsychological testing to detect cognitive impairment in schizophrenia patients is not readily available, but because cognitive deficits in schizophrenia are clinically important it would be useful to detect or at least screen for them in a clinical setting. Unfortunately there are no validated, brief screening instruments for the detection of cognitive impairment in schizophrenia. Nevertheless, the Montreal Cognitive Assessment Test (MoCA) and the Mini-Mental State Examination (MMSE) show promise in this regard. The objective of this study was to compare the results of the MMSE and MoCA in a group of outpatient schizophrenia sufferers to contribute to research into the instruments’ potential usefulness as screening tools for cognitive impairment in schizophrenia.

**Method:**

The Afrikaans versions of the MMSE and MoCA were administered to Afrikaans-speaking adult outpatients. Participants had at least seven years of formal education and had been in partial or full remission for at least 3 months. The MMSE and MoCA scores for each participant were matched and compared using the non-parametric Wilcoxon matched pairs test.

**Results:**

The sample consisted of 30 Afrikaans-speaking outpatients with schizophrenia. The mean MMSE score was 27.17 ± 2.64, and the mean MoCA score was 22.53 ± 3.91. There was a statistically significant difference between participants’ performance on the MMSE and MoCA tests (*p* = 0.000008).

**Conclusion:**

Compared to the MMSE, the MoCA may be a more useful instrument to detect cognitive impairment in patients with schizophrenia. Further studies are required.

## Introduction

Cognitive impairment is a core feature of schizophrenia^1^ that is less amenable to treatment than its two other core features: psychotic symptoms (especially) and negative symptoms.^2^ Schaefer et al. described substantial, generalised cognitive impairment in schizophrenia across multiple domains.^3^ The American Psychiatric Association describes deficits in declarative memory, working memory, language function, executive functions, and slower processing speed.^4^ Cognitive deficits in schizophrenia are significant, because they are common (in up to 75% of patients suffering from schizophrenia^5,6^) and are strongly associated with clinical^7,8^ and functional outcomes.^9,10,11^ The importance of measuring cognitive deficits in schizophrenia is well recognised in that cognitive deficits have become a target for research in clinical trials^10,12^ and furthermore, in 2002, the National Institute of Mental Health established the Measurement and Treatment Research to Improve Cognition in Schizophrenia (MATRICS) initiative to ascertain standards for evaluating outcomes in the treatment of schizophrenia.^13^ Because cognitive deficits in schizophrenia are of such clinical importance, it would be useful to detect them in a clinical setting.

For the detection of cognitive deficits in schizophrenia the ‘gold standard’ is a comprehensive neuropsychological test battery performed by a trained professional.^5,14^ Unfortunately, this method is not readily available in most clinical settings and is consequently not routinely used.^5,14^ A brief screening test for cognitive dysfunction would be the ideal for a busy clinical setting but no such instrument has been specifically validated for schizophrenia^15^ However, because the cognitive deficits of schizophrenia tend to be global,^3^ whilst perhaps targeting specific domains more than others,^4^ the cognitive deficits do not differ much (if at all) from the cognitive domains tested by the Mini-Mental State Examination (MMSE)^16^ and the Montreal Cognitive Assessment Test (MoCA)^17^ even though both were developed for detecting dementia and mild cognitive impairment prior to dementia.^16,17^

The MMSE shows a sensitivity for mild cognitive impairment of only 18% when a cut-off score of 26 out of a possible 30 is taken, whereas the MoCA displayed a sensitivity of 90% for mild cognitive impairment at a cut-off score of 26 out of a possible 30.^17,18^ When a cut-off score of 24 is used for the MMSE, it correlates significantly with dementia at a sensitivity of 0.92 and specificity of 0.96, whereas at a cut-off score of 25 the MMSE continues to correlate significantly with dementia (not mild cognitive impairment) at a sensitivity of 0.97 and specificity of 0.91, in people aged 60 years and older.^18^ Thus, present evidence indicates that the MoCA performs better at screening for mild cognitive impairment, which is exactly what it was designed for.^17^ Although the MMSE is less sensitive than the MoCA for detecting mild cognitive impairment, both tests have a high specificity to do so, namely 87% for the MoCA and between 96 and 100% for the MMSE.^17,18^ The positive predictive value of the MoCA was 89% and the negative predictive value 91% for mild cognitive impairment.^17^ Thus, if the score of the MMSE and MoCA fall below 26 it indicates possible mild cognitive impairment, but because the MoCA is more sensitive, it is more useful for detecting mild cognitive impairment as previously shown by Nasreddine et al.^17^ Because both of these instruments are just screening instruments, further neuropsychological testing would be needed to confirm cognitive impairment in a specific case.

Although the MMSE was initially developed to grade cognitive impairment and to follow the course of cognitive impairment over time, and the MoCA was initially developed for mild cognitive impairment, the MMSE has been widely used since 1975 and the MoCA since 2005 for other indications.^16,17^ The MoCA has become increasingly popular as a cognitive screening instrument in various clinical settings,^19^ because a considerable body of research shows it to be superior to the MMSE in detecting milder forms of cognitive impairment in various patient populations, including those with Parkinson’s disease^20,21^ Huntington’s disease^22^ frontotemporal dementia^23^ and many others.^24,25,26,27^ The superiority of the MoCA to the MMSE for detecting mild cognitive impairment in Parkinson’s disease and frontotemporal dementia was confirmed by a battery of neuropsychological tests.^20,21,23^ A number of studies also compared MMSE- and MoCA results in schizophrenia sufferers which will be further elaborated on in the discussion of the present study.

Most of the studies comparing MoCA and MMSE scores did not validate their outcomes with a battery of tests as was the case in the Parkinson’s disease study above. Thus, these studies took it (as the present study also does) that the MoCA detects mild cognitive deficits irrespective of diagnosis. According to studies thus far, the MoCA seems to be a more useful cognitive screening instrument in schizophrenia than the MMSE.^15,28,29,30^ Nonetheless, screening for cognitive deficits is not the same as diagnosing a syndrome of cognitive impairment. Yet, it may be an indication for referral for a further neuropsychological assessment, which may be important in a specific case, for example, work performance.

Both the MMSE and the MoCA have been translated from English into various languages, including Afrikaans.^31,32^ Although the Afrikaans versions of both the MMSE and MoCA are used in clinical practice to evaluate Afrikaans-speaking patients, neither test has been formally standardised. In everyday practice, the Afrikaans versions of the MMSE and MoCA are used with the assumption that they have been vicariously standardised by the English versions. So, using the Afrikaans versions of the MMSE and MoCA, the objective of this study was to compare the results of the MMSE and MoCA in a group of outpatient schizophrenia sufferers to contribute to research into the instruments’ potential usefulness as screening tools for cognitive impairment in schizophrenia.

To the best of the authors’ knowledge there are few studies comparing results between the MMSE and MoCA in screening for cognitive impairment in schizophrenia. One of them is a South African study by Oosthuizen et al.^33^ However, the aim of that study was completely different to the aim of the present study.

### Methods

This study was approved by the Research Ethics Committee of the Faculty of Health Sciences of the University of Pretoria and the MMSE and MoCA were used with permission. During the study, outpatients of Weskoppies Hospital (WKH) were selected as participants. WKH is a large, governmental, academic, psychiatric hospital in Pretoria. It delivers secondary- and tertiary-level mental health services. Study participants were tested at the outpatient department of WKH and also at two nearby residential facilities.

All participants were adult outpatients suffering from schizophrenia. Participants were selected by convenience sampling whereby every consecutive outpatient suffering from schizophrenia who met the requirements of the study, and who was willing to participate, was included in the study. The requirements for inclusion were: an established DSM-IV TR diagnosis of schizophrenia which had been in remission or partial remission for at least three months; age older than 18 years and younger than 60 years; having the ability to give informed consent; having Afrikaans as first language; and at least seven years of formal schooling. Patients with comorbid medical or psychiatric conditions that could have affected their cognitive performance (such as neurological disorders, major neurocognitive disorder, or substance use disorders) were excluded from the study.

A diagnosis of schizophrenia was established by revision of clinical notes, treating-doctors’ reports, or reports from a facility manager. The reason for selecting outpatients in remission or partial remission was to minimise the effect that disruptive psychotic symptoms may have on test results. The reason for at least seven years of formal education is because it is a requirement for administering the version of the MMSE used in this study.

The first author assessed all participants. The level of education, employment status, living circumstances, marital status, and medication used were recorded. Each participant was assessed with both the MMSE and MoCA. The tests were administered and scored as instructed by the relevant instrument. To avoid measurement error, the MMSE and MoCA were alternated as the first test to be administered to participants. Thus, all odd-numbered participants did the MMSE first and the MoCA second, and all even-numbered participants did the MoCA first and the MMSE second.

The study was planned in consultation with a professional statistician as well as a research consultant from the Department of Information Technology of the University of Pretoria. The MMSE and MoCA scores for each participant were matched and compared using the non-parametric Wilcoxon matched pairs test.^34^

## Results

The Afrikaans versions of the MMSE and MoCA were both administered to 34 Afrikaans-speaking participants. Four participants were later excluded from the study, three because the final diagnosis of the participants turned out to be schizoaffective disorder, and one because of a visual impairment, (which the researchers thought might have unduly impinged on the participant’s performance). Consequently the final statistical analysis was performed on 30 participants who performed both the MMSE and MoCA.

None of the participants suffered from a formal thought disorder that impaired communication. All participants cooperated satisfactorily. Two participants were educated at a special school, but nonetheless acquired seven years of formal training as required by the instructions of the copyright holders of the MMSE. Participants’ demographic data are shown in Table 1, their medication in Table 2, and their test results in Table 3 and Table 4.

**TABLE 1:** Demographic data (*n* = 30).

Demographic	Variable	*N* (%)
Gender	Male	23 (76.67)
	Female	7 (23.33)
Age	Range	21–56 years
	Mean	39.83 ± 9.09 years
	Median	40 years
Marital status	Single	26 (86.67)
	Divorced	4 (13.33)
Education	Grade 7 – Grade 10 or equivalent	6 (20.00)
	Grade 11–12	18 (60.00)
	Tertiary	6 (20.00)
Employment	Unemployed	3 (10.00)
	Disability pension	22 (73.33)
	Labourer	2 (6.67)
	Professional	3 (10.00)
Accommodation	Shelter	1 (3.33)
	Half-Way House	17 (56.67)
	Commune	1 (3.33)
	Full Residential Unit	11 (336.67)

**TABLE 2:** Participants’ medication (*n* = 30).

Drug category	Participants using†
Second generation antipsychotic drugs	29
First generation antipsychotic drugs	8
Anticholinergic drugs	10
Anticonvulsants	7
Antidepressants	20
Sedative hypnotics	9
Beta-blockers	2
Other	1

† Most participants were on more than one drug.

**TABLE 3:** Raw scores obtained with the MMSE and MoCA for participants 1–15.

Variable	Scores														
Participant	1	2	3	4	5	6	7	8	9	10	11	12	13	14	15
MMSE	23	21	28	29	26	29	28	29	24	27	23	30	29	25	29
MoCA	23	17	22	26	20	28	30	28	16	19	16	29	21	17	26

MMSE, Mini-Mental State Examination; MoCA, Montreal Cognitive Assessment Test.

**TABLE 4:** Raw scores obtained with the MMSE and MoCA for participants 16–30.

Variable	Scores														
Participant	16	17	18	19	20	21	22	23	24	25	26	27	28	29	30
MMSE	29	30	30	28	30	29	27	28	28	24	28	26	21	28	29
MoCA	23	25	25	21	27	21	17	23	23	26	20	23	20	22	22

MMSE, Mini-Mental State Examination; MoCA, Montreal Cognitive Assessment Test.

**FIGURE 1:**
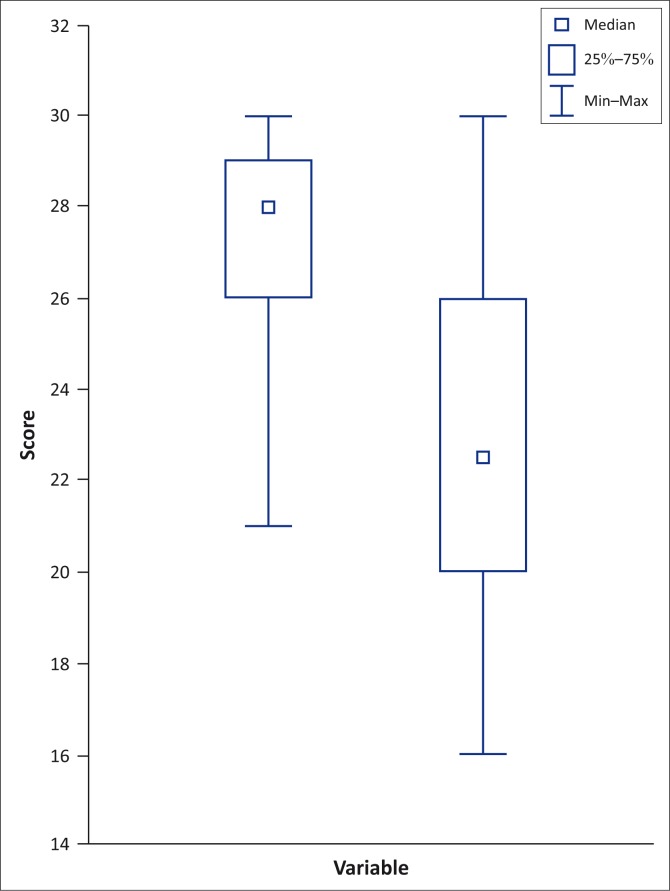
Comparing the Mini-Mental State Examination and Montreal Cognitive Assessment Test scores.

The statistical results of participants’ MMSE tests were as follows: the mean test score was 27.17 ± 2.64 with a median of 28.00. The statistical results of participants’ MoCA tests were as follows: the mean test score was 22.53 ± 3.91 with a median of 22.50. The differences between participants’ MMSE and MoCA test scores differed significantly on the Wilcoxon matched pairs test (*p* = 0.000008 with *p* ≤ 0.05 being significant).^34^ The statistical results are graphically presented in Figure 1.

## Discussion

To the best of the authors’ knowledge, this was the first study to compare the scores of the MMSE and MoCA in an Afrikaans-speaking population of schizophrenia patients. The findings of this study were similar to those of previous studies researching other language groups.^15,28,29,30^ The mean MoCA scores of the present study were significantly lower than the mean MMSE scores found in other studies.^15,28,29,30^ Collectively, these previous findings suggest that the MoCA may be more useful than the MMSE for detecting possible cognitive deficits in schizophrenia patients. Consequently, the results of the present study are in keeping with those studies. Yet, before attributing the significant differences in performance on the MMSE and MoCA in this study to the psychometric properties of the instruments, other reasons for the difference in scores should be explored.

One reason for differences may be because of when the tests were performed in relation to the other. That is, a participant may score better on the MMSE which is always performed first, because of being sharper at the time, whilst when the same person does the MoCA he or she is more fatigued and thus scores poorer. The same scenario can also have a different outcome, namely that the person warms up by doing the MMSE first and thus becomes sharper by the time the MoCA is performed. This problem was addressed by alternating the test that was performed first. At face value the results indicate (Table 3 and Table 4) that irrespective of which test was performed first, the MoCA score was lower. A next possible reason is a learning effect. That is, a participant did the MMSE or MoCA recently and thus performs better on the one than the other. Interestingly, the MMSE does not show a significant learning effect.^16^ The MoCA has excellent test-retest reliability^17^ with very good retest performance even at one month with no significant learning effect.^31^ Although not specifically investigated, it is highly unlikely that either the MMSE or MoCA were administered to study participants in the one month before the study. That is so because neither the MMSE nor MoCA are performed as part of the busy routine follow-up of schizophrenia outpatients at WKH.

Because neither measurement error, nor practice effect reasonably explains participants’ different performances on the MMSE and MoCA, the psychometric properties of the tests appear to be more valuable explanations for the difference. The reason for the MoCA being more useful than the MMSE is explicable in terms of the cognitive domains they evaluate. The MMSE was developed as a method for grading cognitive impairment, and is used to follow up the course of a patient’s cognitive function over time.^36^ The MoCA, on the other hand, was developed to screen for mild cognitive impairment. To do so it tests a wider variety of cognitive functions than the MMSE does. Both the MMSE and MoCA test orientation to time and to place (the MMSE being the more challenging). Both the MMSE and MoCA test the following, with the MoCA being the more challenging in each case: verbal memory, construction, naming, concentration, and repetition^16,17^ The MMSE tests for the following functions which the MoCA does not cover: ideational praxis, reading, and writing; none of which has been reported as impaired in schizophrenia. The MoCA tests the following which the MMSE does not cover: cognitive flexibility, planning, abstraction, working memory, and sustained attention; all of which have been reported as impaired in schizophrenia^2,7,14,37^ and which are not covered by the MMSE. Thus, schizophrenia patients scoring in the unimpaired range on the MMSE may nonetheless have cognitive deficits, which the MMSE does not detect, but which the MoCA identifies. Taking into account the psychometric properties of the MoCA as mentioned in the introduction, a significantly low score (less than 26) on the MoCA is more than just a speculative indication of possible cognitive impairment. It indicates that the MoCA may seriously be considered as a screening tool for cognitive impairment in schizophrenia, and that it needs proper validation studies.

The authors note the following limitations to the study: there is a disproportionate number of male participants, which is an artifact of the convenience sampling method; no other psychometric tests (including the PANSS and neuropsychological tests) were performed; the sample size was modest; and the Afrikaans versions of the MMSE and MoCA have not been standardised. Although the PANSS was not used, these participants were all stable outpatients, and were not suffering from a formal thought disorder that impaired communication. Although the sample size is modest, it is nonetheless large enough to make statistically significant inferences. Finally, it was not formally established whether the MMSE and/or MoCA were recently administered, but, as previously said, such an event is unlikely to explain the findings.

## Conclusion

So far, there is no validated, brief screening instrument for the detection of cognitive impairment in schizophrenia. Previous research indicates that the MoCA may be more useful than the MMSE as a screening instrument for cognitive impairment in schizophrenia. The present study supports this finding. However, the MoCA is only a screening tool, not a diagnostic one. This study, together with previous studies, indicates that the MoCA is a potential screening tool for cognitive impairment in schizophrenia. However, further work is needed to establish the validity of the MoCA compared to the MMSE to detect cognitive impairment in schizophrenia. Further studies are also required to standardise the Afrikaans versions of the MMSE and MoCA.
